# An Overview of Epidemiology of COVID-19 in Macau S.A.R

**DOI:** 10.3389/fpubh.2020.550057

**Published:** 2020-10-30

**Authors:** Sin Man Ieng, Io Hong Cheong

**Affiliations:** Smoke-Free & Healthy Life Association of Macau, Macau, China

**Keywords:** COVID-19, Macau, Greater Bay Area, China, coronavirus (2019-nCoV) outbreak

## Abstract

The Greater Bay Area of southern China has a population of over 71 million people. The area is well-connected with Hubei province, the epicenter of the COVID-19 outbreak. Macau, as the most densely populated city in the world, is very vulnerable to infectious disease outbreaks. Since its return to the sovereignty of China 20 years ago, the city has experienced outbreaks such as severe acute respiratory syndrome (SARS), Swine flu, and COVID-19. At the time of writing, 10 confirmed imported/local transmission cases were recorded. The government undertook measures to attain and then maintain 40 days without new cases. In this article, we report on the 10 confirmed cases and discuss the measures that the Macau Special Administrative Region (S.A.R.) government undertook during the COVID-19 pandemic.

## Introduction

Since the beginning of the COVID-19 pandemic (formerly known as the “novel coronavirus”), the government of the Macau S.A.R has undertaken serious actions to prevent its spread in the community. The Macau S.A.R is located in the megalopolis of the Greater Bay Area, together with the Hong Kong S.A.R. and cities in the Pearl River Delta in Guangdong province. The Greater Bay Area (GBA) has a population of 71 million, the fourth largest bay area in the world after the New York metropolitan area, the San Francisco Bay area, and the Greater Tokyo area. Rapid infrastructure development has meant that the 71 million residents can commute from one city to another within an hour in the area. This region is only 5 h away from Wuhan by high-speed train. There are four international airports in the GBA: Hong Kong, Guangzhou, Shenzhen, and Macau, the region is well-connected. When the severe acute respiratory syndrome (SARS) outbreak hit the region in 2003, the Macau S.A.R. recorded one imported case, while the rest of the region was badly affected by the outbreak. At that time, traffic and economy from mainland China and Hong Kong were restricted by policies and limited infrastructure. The Macau S.A.R. is the most densely populated region in the world [a population of ~670,000 and a population density of 21,717 people per square km (1)] with up to 38 million tourists per year (2), so ineffective infectious disease control can be catastrophic. Despite the potential risk factors for local outbreaks, Macau managed to keep the number of confirmed patients with COVID-19 at 10 for 40 consecutive days (since the last confirmed case on February 4, 2020). Despite its good profile on infectious control, very limited documentation has been published regarding the outbreak response in Macau. This article reviews official publications available in the public domain regarding the COVID-19 pandemic in Macau, with focus on clinical data of the initial 10 confirmed cases and on the strategies that the Macau government adopted to minimize the impact caused by this worldwide pandemic.

## Early Response to the Localized Wuhan Outbreak

On January 3, 2020, the government of the Macau S.A.R. announced its concern of the cluster of cases of unknown pneumonia in Wuhan, Hubei province, China. At that time, there were 44 cases of pneumonia of unknown etiology reported from Wuhan, 11 (25%) of the patients were severely ill (3). In response to this, the government has kept in close contact with the National Health Commission of People's Republic of China from the beginning and has also embraced immediate preventative measures. These measures included measuring the body temperature of arriving air passengers from Wuhan, and also requiring that they complete a health declaration form. The implementation of a health declaration form was also legislated during the 2003 SARS outbreak, arriving visitors were required to declare their personal contact details, travel history in the past 14 days, and health conditions, later the declaration was applied to everyone that entered the Macau S.A.R.

After a cross-departmental meeting, the Health Bureau of Macau issued a Level III Alert on January 5 in response to the outbreak in Wuhan. This system was revised since the 2003 SARS outbreak, the alert meant that there was a moderate risk to public health and activated the following: (1) the Macau Customs Service implemented temperature monitoring at all ports, (2) the Municipal Affairs Bureau executed strict animal import regulations, (3) the Tourism Office kept in close contact with the tourism sector and assisted the Health Bureau in providing infectious control training to the hospitality sector, (4) casinos were obligated to have appropriate equipment to monitor the body temperature of employees and visitors, (5) the Government Information Bureau issued the latest updates on the viral outbreak and information on preventative measures for the public, and (6) paramedics and relevant healthcare-related staff guaranteed the supply of personal protective equipment (PPE) when managing suspicious cases. Visitors and staff to any governmental offices were advised to wear surgical masks and to seek early medical assistance if they experienced fever or respiratory symptoms. Furthermore, government representatives (including the Director of the Health Bureau Center for Disease Control and Prevention) visited Wuhan to inspect the local situation and to reference their approach on diagnosis, treatment, and prevention of the virus. On January 21 2020, the Macau Government established the “Novel Coronavirus Response and Coordination Center.” This 24 h operating center was responsible for coordinating all cross-departmental strategies and polices regarding the prevention, and if necessary, the control of the outbreak (at the time of writing, there were no new confirmed cases of COVID-19 in Macau).

## Reinforcement On Preventative Measures

The first confirmed case of COVID-19 infection in Macau was confirmed on January 22 2020. Thereafter, the government embraced further action to prevent a community outbreak by implementing several public measures. For instance, trained medical personnel were allocated in addition to the existing body monitoring facilities across all the ports and operation hours in these ports were also reduced. The government also urged those who were still in China to return to Macau as soon as possible, preferably within 14 days, to self isolate in case they were asymptomatic. Tour groups from Wuhan were suspended and flights and ferry services to Hong Kong and China were also suspended from early February.

At the same time, the authorities estimated that there were 1,113 visitors from Hubei province entering Macau between the December 1 2019 and January 26 2020. The authority advised these visitors to return to China, whilst those still opting to stay in Macau were quarantined in designated government premises. By January 27, the majority of Hubei visitors had left, leaving behind 371 people (200 of which were from Wuhan) in Macau (4). Thereafter, any Hubei citizens that entered Macau were required to submit a medical certificate stating that they were free from COVID-19 infection.

As the outbreak of COVID-19 continued to progress worldwide, the authorities closely monitored the situation in its neighboring countries. Immigration policies were constantly updated. Since February 24, a mandatory 14 day quarantine was required for visitors who had traveled from South Korea. Subsequently, the same policy applied accordingly to travelers with recent travel history from other high-risk countries (including Italy and Iran) in the past 14 days.

In terms of preventing community transmissions, the Health Bureau and Police Force Department conducted contact tracing of the confirmed cases. These close contacts, once identified, were screened for the COVID-19 virus and quarantined in specific assigned premises for 14 days if initial viral tests were negative. The use of surgical face masks in public were further encouraged and the importance of strict hand-hygiene was widely advertised. The Municipal Affairs Bureau upgraded its routine sanitizing work to a daily basis to ensure that all markets and hawkers' areas were clean. To apply social distancing practices, in early January all public large-scale events were canceled and public libraries, museums, and leisure activity venues were closed down. Citizens were advised to avoid social gatherings and public transport services were reduced. Kindergartens, primary and secondary schools, and all higher education institutions were closed to ensure students were kept safe at home. From January 30 to February 10, 2020, most government non-emergency services were closed down and related civil servants were exempted from work. Later, in February, the Chief Executive of Macau announced for the first time in history that there would be a suspension on the gambling industry, including casinos and associated entertainment facilities, for a period of 15 days.

As part of the early response, the Macau government began actively sourcing medical equipment, including mechanical ventilators, as early as the beginning of January. Moreover, the Health Bureau stressed that their use was to primarily protect all healthcare-related staff from the contagious virus. They ensured that at least a 3 month supply of protective equipment, including PPEs and protective visors were available. To minimize the risk of cross-infection, “Dirty Team” practices were adopted among frontline staff in the government hospitals, for paramedics, and fire brigades.

## Face Masks

To ease public anxiety, after communication with major pharmacies and suppliers in mid-January, the Macau government reassured the public that there would be a sufficient supply of surgical face masks. Since the first imported case of COVID-19, legislation was passed to demand that all staff at casinos and hotels wear surgical face masks. On January 22 2020, the Health Bureau announced a campaign to guarantee that all Macau residents would have an adequate supply of surgical face masks. The government had successfully purchased 20 million face masks which were then distributed to district health centers, these included more than 50 pharmacies and later some non-government organization units. These face masks would be available for purchase at the original cost by Macau residents and non-resident workers, in a batch of 10 face masks at a price of MOP$0.80 each (approximates to USD$0.10). Each person would be able to purchase 10 face masks every 10 days. The government put enormous effort into searching for suppliers of face masks worldwide and ensuring that the campaign would continue during the pandemic period, including chartering flights to transport these masks ensuring there was a sufficient supply for the public. On February 3, the Macau Transport Bureau (DSAT) announced that all public transportation drivers and passengers would be required to wear face masks when embarking public transport vehicles (5). Furthermore, people were strongly advised to wear face masks in public.

## The 10 Cases of COVID-19 in Macau

Macau had its first confirmed case of COVID-19 2 weeks after the level III alert was announced. A confirmed case is defined as a person with laboratory confirmation of COVID-19 infection, irrespective of clinical signs and symptoms (6). On February 2, there were another nine new confirmed cases. Table 1 illustrates the demographics and clinical course of these 10 confirmed cases in Macau. Out of the 10 cases, seven were tourists from Wuhan and three were Macau residents. The first eight cases were classified as imported cases whilst the last two were either imported or local transmission cases since these two patients had both recent travel history to China and contact with high risk groups. Transmission classification is based on the WHO analysis of available official data and may include: (a) community transmission—where it is unable to relate the confirmed cases through chains of transmission for a large number of cases, (b) local transmission—which indicates locations where the source of infection is within the reporting location, and (c) imported cases—which indicates cases that have been acquired outside the location of reporting.

**Table 1:** Demographics and clinical course of the first 10 confirmed cases in Macau.

**Case**	**Date of diagnosis**	**Age**	**Region of residence**	**Background**	**Recent travel history**	^*^Transmission classification	**Clinical complications**	**Clinical outcome**	**Duration of hospitalization**
1	2020/1/22	45–60	Wuhan	Tourist	Wuhan (China), Zhuhai (China), Macau	Imported	None	Cured	15 days
2	2020/1/21	60+	Wuhan	Tourist	Wuhan (China), Zhuhai (China), Macau	Imported	None	Cured	28 days
3	2020/1/26	45–60	Wuhan	Tourist	Wuhan (China), Zhuhai (China), Macau	Imported	Hypoxemia, received steroid therapy	Cured	19 days
4	2020/1/26	30–45	Wuhan	Tourist, mother of case 6	Wuhan (China), Hong Kong, Macau	Imported	None	Cured	21 days
5	2020/1/26	15–30	Wuhan	Tourist	Wuhan (China), Zhuhai (China), Macau	Imported	None	Cured	17 days
6	2020/1/27	15–30	Wuhan	Tourist, son of case 4	Wuhan (China), Hong Kong, Macau	Imported	None	Cured	22 days
7	2020/1/27	60+	Wuhan	Tourist	Wuhan (China), Guangzhou (china), Zhuhai (China), Macau	Imported	None	Cured	28 days
8	2020/2/2	60+	Macau	Resident	Zhuhai (China), ZhongShan (China), Macau	Imported	Pneumonia at presentation, hypoxemia, received steroid therapy	Cured	34 days
9	2020/2/4	15–30	Macau	Resident, history of contact with case 8	Macau	Imported/local	None	Cured	29 days
10	2020/2/4	45–60	Macau	Resident	Guangzhou (China), Macau	Imported/local	None	Cured	24 days

* *Transmission classification is based on WHO analysis of available official data and may include: (a) community transmission—where it is unable to relate the confirmed cases through chains of transmission for a large number of cases, (b) local transmission—which indicates locations where the source of infection is within the reporting location, and (c) imported cases—which indicates cases that have been acquired outside the location of reporting. A publicly accessible dataset was analyzed in this study. This can be found here: https://www.ssm.gov.mo/apps1/PreventCOVID-19/en.aspx#clg17046*.

During the clinical course of these 10 confirmed cases, all patients were treated in an isolation ward in the hospital. According to information provided by the Novel Coronavirus Response and Coordination Center, all 10 patients received anti-viral treatment. A total of (20%) experienced hypoxemia during hospitalization and required the used of steroid therapy. None of them needed intubation and mechanical ventilation, mortality rate was zero. All (100%) patients were cured and discharged. Discharge criteria was in accordance with the “Diagnosis and Treatment Protocol for Novel Coronavirus Pneumonia” published by the National Health Commission, China, that includes the absence of fever, the clinical resolution of symptoms, improvement in imaging tests of the chest, and repeatedly negative nucleic acid results in nasopharyngeal swabs. All seven patients from Wuhan left for China on the same day of discharge. Meanwhile the three local Macau residents were discharged to a designated venue for a 14 day quarantine during the recovery period, they were to be tested again for the virus at regular time intervals. This extended quarantine standard is twice as long when compared to the standards advised by the National Health Commission in China. The extended quarantine period was to ensure that the patients' virology tests would not turn positive again before they returned to the community.

## COVID-19 in Neighboring Regions

The pandemic progression of COVID-19 in places neighboring Macau appeared to develop in a similar pattern and magnitude. Nearby regions that share comparable age and gender distribution, ethnic diversity, cultural background, and economic status include the Hong Kong S.A.R., Taiwan, and Singapore. Table 2 shows a summary of the epidemiology of COVID-19 in these regions in the time frame of January to March/April 2020. As illustrated, these regions all had a relatively low incidence and mortality rate. Similar to Macau, given the high population density and close geographical connections to mainland China, these regions were highly vulnerable to a community-wide outbreak. In other overseas countries, the first wave of COVID-19 case importation was usually followed by widespread community transmission. However, these did not appear to be the case here. These regions (and Macau) all demonstrated a higher proportion of imported cases. This can be explained by the execution of early public interventions in each of these regions—namely city-wide lockdown, social distancing, and voluntary facemask-wearing in the community. These interventions, in fact, have later been identified to have had a positive impact on public health by several studies (13, 14).

**Table 2:** Comparison of epidemiology and demographics of cases of COVID-19 in Macau with neighboring regions during the initial phases of the pandemic.

	**Macau S.A.R (up to March 14 2020)**	**Hong Kong S.A.R (8, 9) (up to April 19 2020)**	**Taiwan (10) (up to March 18 2020)**	**Singapore (11) (up to March 31 2020)**
Population (12)	0.6 million	7.5 million	23.8 million	5.9 million
Population density (per km^2^)	21,717	7,140	673	8,358
Percentage of urbanity	100%	100%	78.9%	100%
Incidence of COVID-19	10	1025	100	926
**Mode of transmission:**				
Import	10 (100%)	604 (59%)	71 (72%)	525 (57%)
Non-import	0	421 (41%)	29 (28%)	401 (43%)
Number of mortality	0	2	3	3

*Numbers in () represent percentage of cases of COVID-19 classified by mode of transmission*.

## Discussion

Under the one-country two-system framework, the S.A.R. enjoys a high degree of autonomy including its own immigration customs border and policies. In the 2003 SARS outbreak, Macau had only one imported case. Entry to the Macau S.A.R. at the time was restricted to only a small number of tour groups or transiting tourists from the mainland. Connections to Hong Kong were limited to helicopters and ferries, air traffic was also relatively low. The government at the time implemented the following policies to control the outbreak: they established an inter-departmental coordination workgroup, health declaration, body temperature measures at entry ports, specialized fever areas at emergency sections in hospitals, a hand wash and household disinfection campaign, and an infectious disease reporting system between Hong Kong and Guangdong province.

Some of the policies still remain active today, the monitoring of body temperature at major ports and communication within the region are still in place. After the SARS outbreak, China opened a free individual traveler scheme for their citizens to visit the two S.A.R.s as an economy stimulus package. The policy attracts millions of tourists every year, and the measures were deployed again in the successful control of the swine flu outbreak. Since then, Macau has gradually integrated into the region. Over the past 17 years, the area has rapidly developed into a world class megalopolis and infrastructure, Macau has received 21 times more tourists than in 2003, the original measures were inadequate for the increasing capacity.

The government upgraded some of the existing measures, and introduced new measures to contain the new virus. This included digitizing the health declaration form and integrating with the national immigration network to verify the travel history of visitors. Public health promotion was also adopted on social media. Information on places and transport routes used by confirmed individuals could be released and circulated in a short time frame. The introduction of the mask guarantee scheme reassured the public and maintained public confidence in the government, the masks were distributed with the help of a centrally registered network to provide a real-time track record on the stock. The government also operated a closed management system for those who had returned from high risk regions, individuals were quarantined in designated venues. In addition to this, the government also provided social care support to those who were quarantined. These measures heightened the confidence of the public in the government with the added benefit of keeping the public informed and ensuring that they learnt the correct response.

## Conclusion

At the time of writing, no new cases of COVID-19 infections have been reported in Macau for the past 40 days (from February 4 to March 14, 2020). Import transmission was minimized by an early response by the government and the isolation of potential infectious sources by identifying visitors from high incidence areas. Many of the policies abided by the WHO's guidance on protective measures—including promoting hand hygiene and social distancing. Moreover, being a densely populated region, the government understood that social distancing is not always possible. Therefore, the government advised the use of face masks, and this seems to have been effective in limiting community transmission.

Up until this paper was submitted, there had been 34 new cases of confirmed COVID-19 infections in Macau (7), all of which were imported cases. These imported cases were mainly overseas students and residents returning to Macau from Europe or North America, and this corresponded to the increasing incidence of COVID-19 in those parts of the world. Nonetheless, the Macau government continued to pursue effective policies to combat this pandemic—which can be summed up in the government's message to the public: “Let's all persist: avoid crowd gathering, wash hands frequently, wear a mask properly, declare health conditions, reduce leaving Macau.” The region has a lot of logistic, medical, and economic developments planned. In the future, there should be more resources given to a disease control system within the region, these development should be synchronized along with the economic development in the region.

## Data Availability Statement

Publicly accessible dataset were analyzed in this study. These can be found here: https://www.ssm.gov.mo/apps1/PreventCOVID-19/en.aspx#clg17046.

## Author Contributions

SI contributed to the current policy and confirm cases review and the writing of the manuscript. IC contributed background and previous experience review. All authors contributed to the article and approved the submitted version.

## Conflict of Interest

The authors declare that the research was conducted in the absence of any commercial or financial relationships that could be construed as a potential conflict of interest.
